# Does Aprotinin Reduce the Blood Loss after Total Hip Arthroplasty? A Meta‐analysis of Randomized Controlled Trials

**DOI:** 10.1111/os.12459

**Published:** 2019-04-26

**Authors:** Jin Xu, Xin‐long Ma

**Affiliations:** ^1^ Department of Orthopaedic Surgery Tianjin Hospital Tianjin China

**Keywords:** Aprotininl, Blood loss, Meta‐analysis, Total hip arthroplasty

## Abstract

We perform a meta‐analysis from published randomized controlled trials to assess the efficacy and safety of aprotinin in total hip arthroplasty (THA). The following electronic databases were searched: PubMed (1966 to December 2018), EMBASE (1974 to December 2018), the Cochrane Library (1974 to December 2018), and Web of Science (1990 to December 2018). We also used Google Search to search for more potentially eligible studies up to December 2018. The methodological quality of the included studies was assessed independently by the two reviewers described by the Cochrane Collaboration for Systematic Reviews. Data analysis was performed with STATA13.0. Four randomized controlled trials were included in the meta‐analysis. Our study indicated that intravenous aprotinin was associated with improved outcomes in terms of total blood loss, hemoglobin decline, and transfusion rates. There was no significant difference regarding the length of stay and the risk of deep venous thrombosis and pulmonary embolism. Intravenous aprotinin was effective and safe to use in reducing total blood loss after total hip arthroplasty. Further high‐quality studies are required to confirm the conclusion.

## Introduction

Total hip arthroplasty (THA) is associated with large total blood loss, reported to range from 260 to 780 mL^1^, ^2^, ^3^. Thus, blood management after THA is an important issue. Many kinds of techniques, including autologous fibrin tissue and aminocaproic acid, have been used to decrease bleeding and blood transfusion requirements after major orthopaedic surgery^4^, ^5^, ^6^. Tranexamic acid (TXA) is a medication used to treat or prevent excessive blood loss from major trauma, postpartum bleeding, and surgery. Barrachina *et al*.^7^ reported that a single preoperative dose of TXA or two infusions of a lower dose, preoperatively and then 3 h after the start of surgery, resulted in lower blood loss during the first 2 days after surgery and less need for blood transfusion, with good levels of safety. Chen *et al*.^8^ reported that topical TXA could significantly reduce total blood loss, drainage loss, and transfusion rates, and decrease hemoglobin levels following THA, without increasing the risk of venous thromboembolisms. However, TXA, as an anti‐fibrinolysis, may potentially increase the risk of thrombotic complications.

Aprotinin is a small protein bovine pancreatic trypsin inhibitor (BPTI) that inhibits trypsin and related proteolytic enzymes. Aprotinin acts to inactivate free plasmin but with little effect on bound plasmin, whereas the lysine analogues are designed to prevent excessive plasmin formation by fitting into plasminogen’s lysine‐binding site to prevent the binding of plasminogen to fibrin. Previous studies have reported that intravenous aprotinin is associated with improved outcomes in terms of blood loss^9^, ^10^. However, the efficacy of aprotinin remains controversial. Therefore, we perform a meta‐analysis from published randomized controlled trials to assess the efficacy and safety of aprotinin in THA.

## Methods

### *Search Strategy*

The following electronic databases were searched: PubMed (1966 to December 2018), EMBASE (1974 to December 2018), the Cochrane Library (1974 to December 2018), and Web of Science (1990 to December 2018). We also used Google Search to search for more potentially eligible studies up to December 2018. The following key words were used for these databases above: (total hip arthroplasty OR total hip replacement OR TKR) AND (aprotinin). There were no language or geographical restrictions.

### *Inclusion and Exclusion Criteria*

The inclusion criteria for these studies were: (i) people: patients with THA; (ii) intervention: intravenous aprotinin; (iii) comparison: placebo; (iv) outcome measures: at least one of the following outcome measures was reported: total blood loss, hemoglobin decline, transfusion requirements, opioid consumption, length of hospitalization, and adverse events; and (iv) study design: randomized controlled trial (RCT). In contrast, revision or simultaneous bilateral THA were excluded. Two of the reviewers independently scanned the titles and abstracts to search for potential studies, and then finally obtained the eligible studies for inclusion based on further review of full texts. Disagreements were resolved by consensus after discussion, or a third reviewer was consulted if necessary.

### *Assessment of Methodological Quality*

The methodological quality of the included studies was assessed independently by the two reviewers based on the Cochrane Collaboration for Systematic Reviews. The six items of sequence generation, allocation sequence concealment, blinding, incomplete outcome data, selective outcome reporting, and other potential risks were considered to be meaningful for an evaluation index. The methodological quality of each RCT study was assessed as low (low risk of bias), high (high risk of bias), and unclear (unclear risk of bias). The two reviewers independently used the modified Jadad scale to assess the risk of bias of the included studies. Studies obtaining 4 or more points (up to 8 points) were considered to be of high quality, and any disagreement was resolved by consensus after discussion, and, if necessary, the third reviewer was consulted.

### *Data Extraction*

Two reviewers independently extracted outcomes from each RCT study. The data includes: authors, publication year, patients, age, body mass index (BMI), the intervention method, transfusion criterion, and the method of deep venous thrombosis (DVT) prophylaxis and screening. The primary outcomes in this meta‐analysis were transfusion requirements, DVT and pulmonary embolism (PE). We also used blood loss, drainage volume, and length of stay as the secondary outcomes between the two groups. Missing or insufficient information were obtained by emailing the original author. Disagreement was resolved by consensus after discussion, and, if necessary, the third reviewer was consulted.

### *Data Analysis*

Data analysis was performed with STATA13.0 (StataCorp, College Station, Texas, US). A random‐effects model was adopted to pool the results. The risk ratio (RR) was used as a summary statistic for dichotomous variables and the mean difference (MD) was used to analyze continuous variables. Both were reported with 95% confidence intervals (CI); a *P*‐value of 0.05 was used as the level of statistical significance. Statistical heterogeneity between trials was evaluated using χ^2^ and *I* ^2^ tests (*I* ^2^: 0%–30% was considered homogeneous, 30%–60% was considered moderately heterogeneous, and > 60% was considered substantially heterogenous), with significance set at *P* < 0.10.

## Results

### *Characteristics of Included Studies*

**Figure 1:**
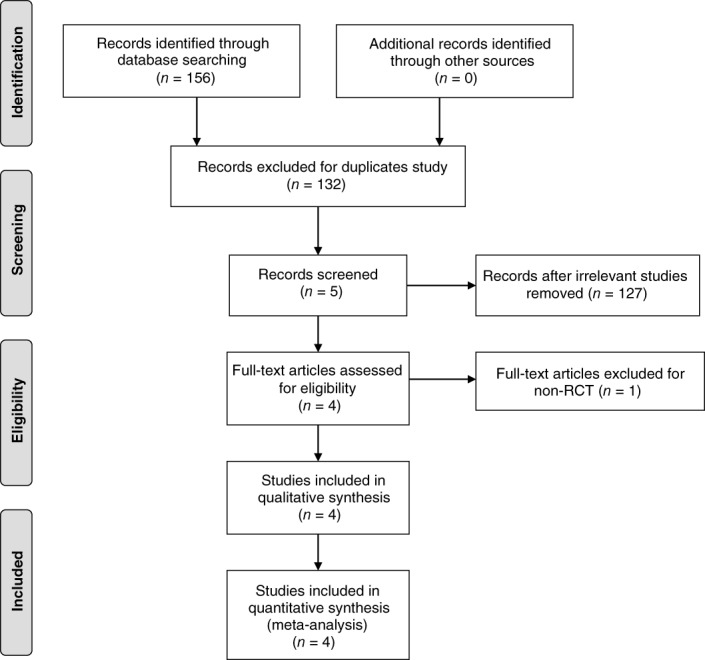
Flow chart of study selection. RCT, randomized controlled trial.

A summary of the study selection process is presented in Fig. 1. Our search identified 156 records. A total of 132 citations were discarded because they were duplicates or did not fit the eligibility criteria. After full‐text verification of the remaining 24 articles, 4 studies^11^, ^12^, ^13^, ^14^ were included in the meta‐analysis.

### *Study Characteristics*

All included RCT were published between 2010 and 2018. The sample size ranges from 30 to 369. In the intervention group, intravenous aprotinin was used, and control groups received placebo or nothing. The duration of follow‐up ranges from 1 to 6 months. The main characteristics of included studies are reported in Table 1.

**Table 1:** Cohort characteristics

Studies	Cases (A/C)	Mean age (A/C)	Female patients (A/C)	Surgical procedure	Anesthesia method	TXA intervention	Transfusion trigger	Follow‐up (months)
Murkin (2000)	29/24	67/66	14/12	THA	General anesthesia	A: Aprotinin: 2 × 10^6^ KIU C: placebo	Hemoglobin < 7 g/dL	1
Langdown (2000)	30/30	62/65	20/13	THA	Spinal anesthesia	A: Aprotinin: 1.5 × 10^6^ KIU C: placebo	Hemoglobin < 7 g/dL	2
Ray (2005)	15/15	72/69	10/9	THA	General anesthesia	A: Aprotinin: 2 × 10^6^ KIU C: placebo	Hemoglobin < 7 g/dL	3
Petsatodis (2006)	25/25	58/60	12/14	THA	Spinal anesthesia	A: Aprotinin: 2 × 10^4^ KIU C: placebo	Hemoglobin < 7 g/dL	1

A, aprotinin group; C, control group; THA, total hip arthroplasty; TXA, tranexamic acid.

### *Risk of Bias in Included Studies*

**Figure 2:**
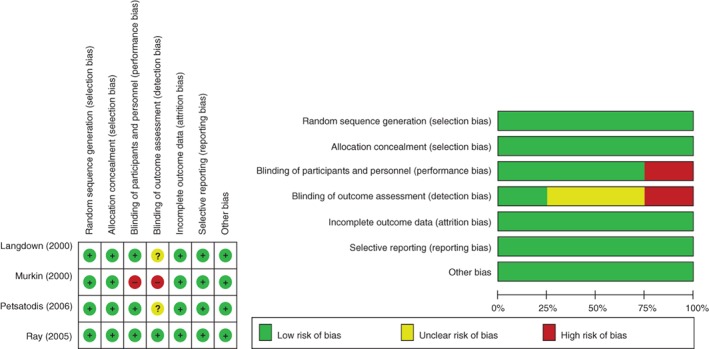
Risk of bias summary.

The authors’ judgments about each risk of bias item for each included study are presented in Fig. 2. The percentages of performance bias were highest because none of 5 studies report the blinding of participants and personnel. Only one study did not clearly report the procedures of randomization and allocation concealment; hence an unclear risk bias was considered across this study. All studies clearly reported the blinding of outcome assessment. Only two studies clearly reported the reasons for lost follow‐up.

### *Outcome of Meta‐analysis*

#### *Total Blood Loss*

**Figure 3:**
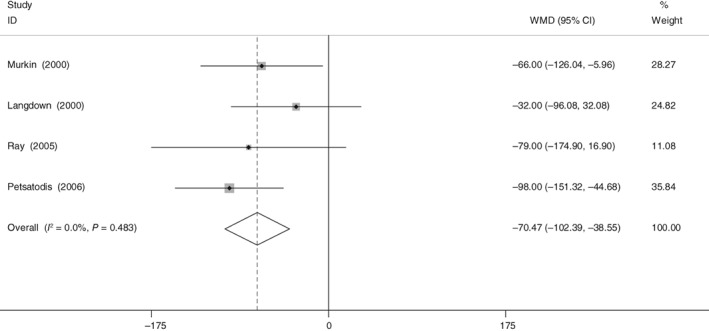
Forest plot diagram shows the proportion of total blood loss.

Four RCT reported the total blood loss after THA. There was no significant heterogeneity (χ^2^ = 2.46, df = 3, *I* ^2^ = 0%, *P* = 0.48). The present meta‐analysis indicated that aprotinin could significantly reduce total blood loss (WMD = −70.470, 95% CI: −102.391 to −38.550, *P* < 0.001; Fig. 3).

#### *Hemoglobin Decline*

**Figure 4:**
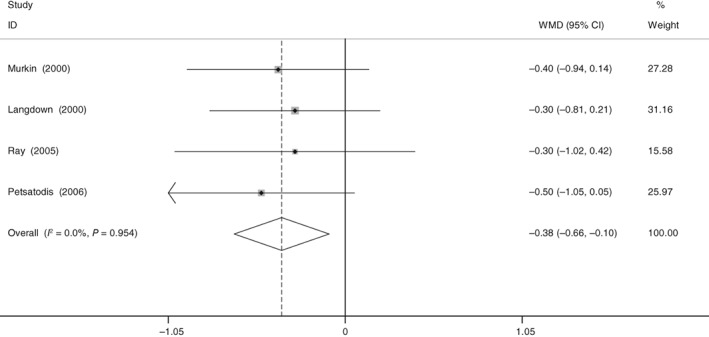
Forest plot diagram shows the proportion of hemoglobin decline.

All studies showed the hemoglobin decline after THA. No significant heterogeneity (χ^2^ = 0.33, df = 3, *I* ^2^ = 0%, *P* = 0.95) was found. Our meta‐analysis demonstrated that aprotinin could significantly reduce hemoglobin decline (WMD = −0.379, 95% CI: −0.662 to −0.097, *P* = 0.009; Fig. 4).

#### *Transfusion Rates*

**Figure 5:**
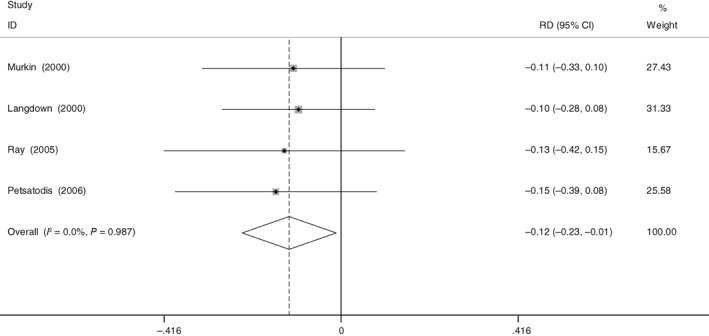
Forest plot diagram shows the proportion of transfusion rate.

Four of the selected studies provided eligible data for the pooled analysis of transfusion rates. The results demonstrated that there was significance of the transfusion rates between the aprotinin group and the control group (RD = −0.122, 95% CI: −0.233 to −0.012, *P* = 0.030; Fig. 5). Evidence showed low heterogeneity, and no significant heterogeneity was found (χ^2^ = 0.14, df = 3, *I* ^2^ = 0%, *P* = 0.987).

#### *Length of Stay*

**Figure 6:**
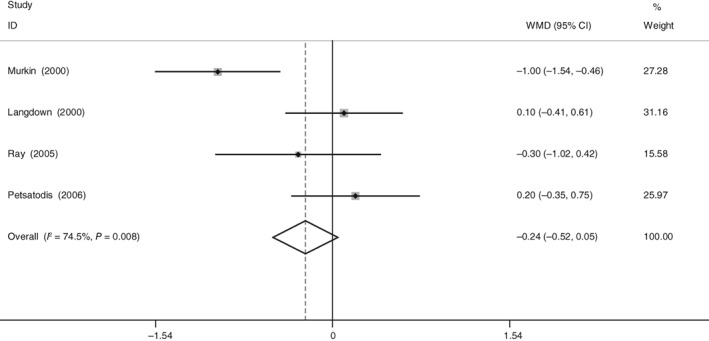
Forest plot diagram shows the proportion of length of stay.

Four RCT reported the length of stay after THA. There was significant heterogeneity (χ^2^ = 11.77, df = 3, *I* ^2^ = 74.5%, *P* = 0.008). The present meta‐analysis indicated that there was no significant difference between groups regarding length of stay (WMD = −0.236, 95% CI: −0.519 to 0.046, *P* = 0.101; Fig. 6).

#### *Deep Venous Thrombosis*

**Figure 7:**
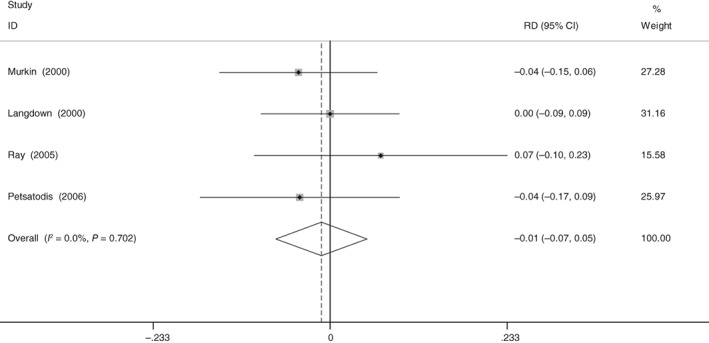
Forest plot diagram shows the proportion of deep venous thrombosis.

All included studies provided data on the incidence of DVT. There was no significant heterogeneity (χ^2^ = 1.42, df = 3, *I* ^2^ = 0%, *P* = 0.702). The present meta‐analysis indicated that aprotinin did not increase the risk of deep venous thrombosis (RD = −0.011, 95% CI: −0.071 to 0.049, *P* = 0.710; Fig. 7).

#### *Pulmonary Embolism*

**Figure 8:**
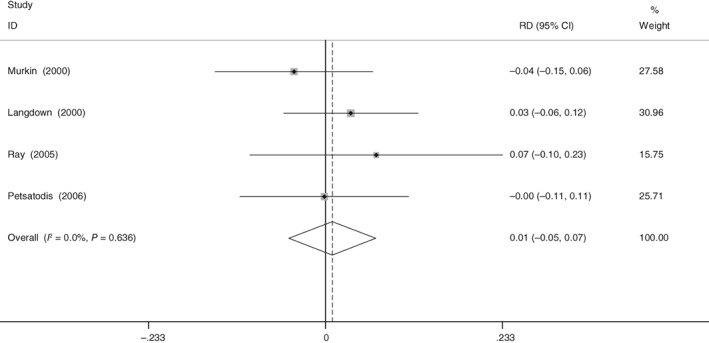
Forest plot diagram shows the proportion of length of hospitalization pulmonary embolism.

Four included RCT showed the data on the incidence of PE. No significant heterogeneity was identified (χ^2^ = 1.42, df = 3, *I* ^2^ = 0%, *P* = 0.702) and a fixed effect model was used. The present meta‐analysis indicated that aprotinin did not increase the risk of PE (RD = 0.009, 95% CI: −0.048 to 0.066, *P* = 0.760; Fig. 8).

## Discussion

Blood management after major orthopaedic surgery has been discussed for several decades. To our knowledge, this is the first meta‐analysis from RCT to compare aprotinin and control groups for reductions in blood loss after THA. The most interesting finding of the present meta‐analysis was that intravenous aprotinin could significantly reduce the total blood transfusion rates and hemoglobin decline. No significant difference was found in terms of length of stay and the risk of DVT or PE.

Aprotinin has been routinely used in THA over the past decade and has the advantage of reducing blood loss, the need for blood transfusion, and hospitalization costs. Aprotinin can be administrated intravenously, topically, and orally^15^, ^16^. All included studies used intravenous application, so we did not perform the subgroup analysis for other kinds of use. Murkin *et al*. demonstrated that intravenous aprotinin was associated with improved outcomes for blood management^13^; however, Hayes *et al*. reported that a single 2 million KIU bolus dose of aprotinin does not reduce blood loss or transfusion requirements. Based on the controversy of the published studies, we performed this meta‐analysis of recently published RCT and found that intravenous aprotinin was associated with a significant reduction in total blood loss after THA.

Blood transfusions are common following total joint arthroplasty. An estimated 2–4 units of blood required for transfusions after THA^17^, ^18^, ^19^. Allogenic blood transfusion is associated with numerous adverse reactions, including anaphylactic reactions, infectious diseases, as well as metabolic disorders prolonging the length of hospital stay, and resulting in serious medical costs^20^, ^21^. Fleischmann *et al*.^22^ showed that the average blood transfusion requirement was 370 mL in aprotinin groups compared to 680 mL in control groups. However, there remains controversy regarding the efficacy of intravenous aprotinin in THA. The present meta‐analysis indicated that the use of aprotinin results in a significant reduction in transfusion rates.

Although aprotinin is associated with a potential risk of thrombosis, published articles have not identified an increased risk of thromboembolic events. Several articles have even reported that aprotinin reduces the risk of thrombosis events by reducing transfusion requirements. The present meta‐analysis showed that there was no significant difference in terms of DVT and PE between groups. This low incidence of thromboembolic events could be associated with reasonable prevention of thrombosis, including low‐molecular‐weight heparin, aspirin, enoxaparin, and clinical functional exercise.

Several limitations of the present meta‐analysis should be noted. First, the small sample size might have affected the significant difference between the groups. Second, the possibility of publication bias existed for the limited number of included trials. Third, some important outcomes such as functional recovery and pain scores were not discussed. Despite the limitations outlined above, this was the first meta‐analysis from RCT to evaluate the efficacy and safety of aprotinin for reducing blood loss after THA. More high quality RCT are required.

### *Conclusion*

Intravenous aprotinin was effective and safe to use in reducing total blood loss after total hip arthroplasty. Further high‐quality studies are required to confirm the conclusion.
